# Giant cell myocarditis treated with long-term multi-modal care with multiple mechanical circulatory support: case report

**DOI:** 10.1093/omcr/omag110

**Published:** 2026-07-08

**Authors:** Motohiro Shingu, Nishikawa Tatsuya, Yuya Oga, Hibiki Kadohara, Yuto Osumi, Kenta Ishibashi, Mana Hiraishi, Mitsuo Kinugasa, Yasutaka Hirayama, Kinta Hatakeyama, Koichi Tamita

**Affiliations:** Department of Cardiovascular Medicine, Akashi Medical Center, 743-33 Yagi, Ohkubo-cho, Akashi, Hyogo 674-0063, Japan; Department of Cardiovascular Medicine, Akashi Medical Center, 743-33 Yagi, Ohkubo-cho, Akashi, Hyogo 674-0063, Japan; Department of Cardiovascular Medicine, Akashi Medical Center, 743-33 Yagi, Ohkubo-cho, Akashi, Hyogo 674-0063, Japan; Department of Cardiovascular Medicine, Akashi Medical Center, 743-33 Yagi, Ohkubo-cho, Akashi, Hyogo 674-0063, Japan; Department of Cardiovascular Medicine, Akashi Medical Center, 743-33 Yagi, Ohkubo-cho, Akashi, Hyogo 674-0063, Japan; Department of Cardiovascular Medicine, Akashi Medical Center, 743-33 Yagi, Ohkubo-cho, Akashi, Hyogo 674-0063, Japan; Department of Cardiovascular Medicine, Akashi Medical Center, 743-33 Yagi, Ohkubo-cho, Akashi, Hyogo 674-0063, Japan; Department of Cardiovascular Medicine, Akashi Medical Center, 743-33 Yagi, Ohkubo-cho, Akashi, Hyogo 674-0063, Japan; Department of Cardiovascular Medicine, Akashi Medical Center, 743-33 Yagi, Ohkubo-cho, Akashi, Hyogo 674-0063, Japan; National Cerebral and Cardiovascular Center, 6-1 Kishibe-Shimmachi, Suita, Osaka 564-8565, Japan; Department of Cardiovascular Medicine, Akashi Medical Center, 743-33 Yagi, Ohkubo-cho, Akashi, Hyogo 674-0063, Japan

**Keywords:** Giant cell myocarditis, IMPELLA, ECMO, immunosuppressive therapy

## Abstract

We report the case of a 71-year-old woman with giant cell myocarditis presenting as acute heart failure and cardiogenic shock (LVEF 15%). She required mechanical ventilation, IMPELLA CP (Abiomed, Danvers, MA), veno-arterial extracorporeal (VA-ECMO), and continuous renal replacement therapy. Endomyocardial biopsy confirmed giant cell myocarditis, and treatment with corticosteroids, mycophenolate mofetil, and tacrolimus was initiated. Following multi-modal support and immunosuppressive therapy, left ventricular function recovered to 54%, allowing withdrawal of mechanical circulatory support and hemodialysis. However, the patient ultimately died on the 111th day due to the long treatment of cytomegalovirus infection and septic shock from catheter-related blood stream infection. Optimal immunosuppressive regimens remain undefined, so this case highlights successful recovery of cardiac function with intensive multi-modal management.

## Introduction

Giant cell myocarditis is a rare disease associated with poor prognosis. It often leads to congestive heart failure, refractory ventricular arrhythmias, and cardiogenic shock [1, 2]. We report a case of giant cell myocarditis that was treated by multi-modal treatment including heart pump (IMPELLA, Abiomed, Danvers, MA), extracorporeal membrane oxygenation (ECMO) and a triple immunosuppressive regimen.

## Case report

**Figure 1:**
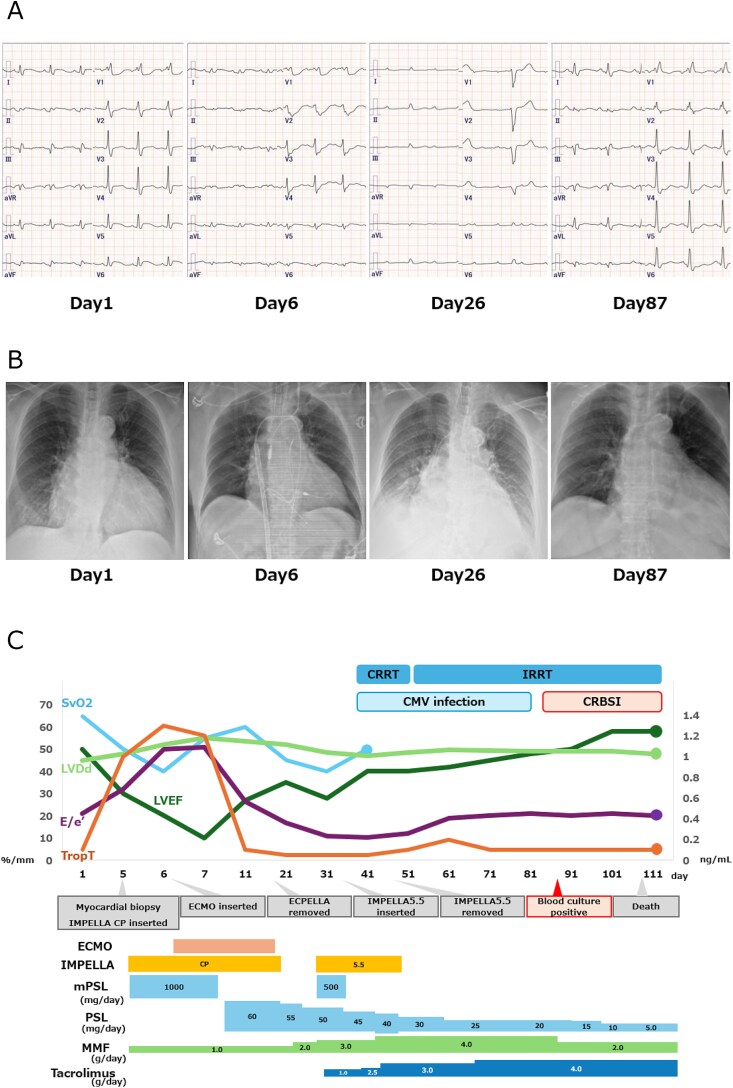
(A): The voltage of the electrocardiograms decreased with worsening giant cell myocarditis but was finally improved by multidisciplinary treatment. (B): In chest radiography, the pulmonary edema was getting worse and the clinical course of myocarditis markings were observed. Finally, the findings were improved with multidisciplinary treatment. (C): After the administration of mycophenolate mofetil, the serum troponin T levels gradually decreased along with a gradual improvement in the left ventricular ejection fraction. CMV; cytomegalovirus, CRBSI; catheter-related blood stream infections, CRRT; continuous renal replacement therapy, ECMO; extracorporeal membrane oxygenation, IRRT; intermittent renal replacement therapy, MMF; mycophenolate mofetil, mPSL; methylprednisolone, PSL; prednisolone, SvO2: Mixed venous oxygen saturation.

**Figure 2:**
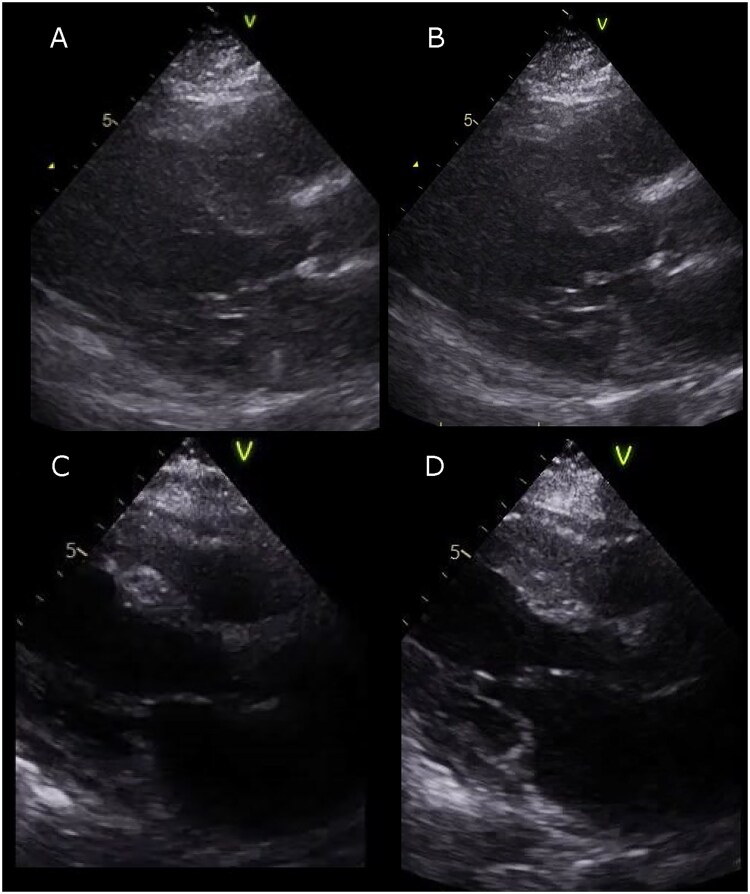
On admission (A: End-systolic, B: End-diastolic) shows severely depressed left ventricular (LV) systolic dysfunction with an LV ejection fraction (LVEF) as low as 38% in transthoracic echocardiography. Finally, LVEF recovered to 54% (C: End-systole, D: End-diastole).

**Figure 3:**
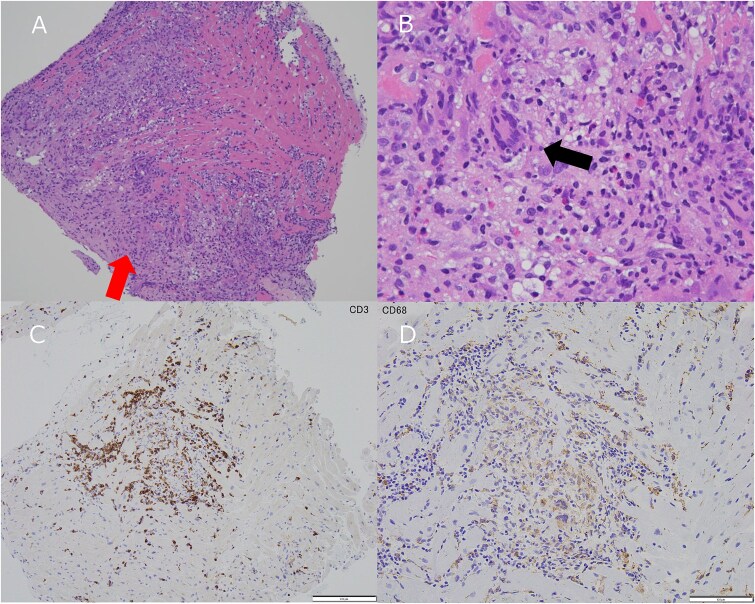
Histological findings of Hematoxylin and eosin staining obtained from the right ventricular septal (A: ×40, B: ×100). Figure 3-A shows severe myocardial contusion (arrow). Figure 3-B shows the infiltration of lymphocytes, eosinophils and giant cells (arrow). The invasion of CD3 positive T cells (C: ×40) and CD68 positive macrophage (D: ×40) was consistent with the infiltration of giant cells.

A 71-year-old woman presented to our emergency department with dyspnea and a one-week history of productive cough. On arrival, she was afebrile with blood pressure of 113/69 mmHg, a heart rate of 103/min, a respiratory rate of 23/min, and a SpO₂ of 95% (ambient air). On admission, electrocardiogram showed a widened QRS duration of 112 msec, and an incomplete right bundle branch block (Fig. 1A). Laboratory evaluation revealed elevated levels of creatine kinase (239 IU/L), C-reactive protein (4.51 mg/dl), serum creatinine (0.70 mg/dl) and high-sensitivity troponin T (1.300 ng/ml). Chest radiography demonstrated bilateral infiltrates with central predominance (Fig. 1B). No coronary artery stenosis was seen in catheter-based coronary angiography. Transthoracic echocardiography revealed left ventricular (LV) systolic dysfunction with an ejection fraction (LVEF) of 38% (Fig. 2). The patient was managed with furosemide, dobutamine and non-invasive positive pressure ventilation for acute heart failure. However, her symptoms worsened with persistently rising troponin T levels (Fig. 1C). On the 5th day, LVEF dropped to 20% with ongoing mild pericardial effusion. As there was suspicion of fulminant myocarditis, we performed a myocardial biopsy and implanted an IMPELLA CP device. During the procedure, CAVB developed, which necessitated the insertion of a temporary pacemaker. We performed a 3-day course of intravenous methylprednisolone (1000 mg daily), and initiated immunosuppressive therapy with mycophenolate mofetil (1000 mg daily). In anticipation of infection prophylaxis, we tested for HBs antigen, HBs antibody, HBc antibody, and T-SPOT, all of which was negative. Additionally, sulfamethoxazole/trimethoprim was prescribed in an effort to prevent pneumocystis pneumonia. For mycophenolate mofetil, routine monitoring of levels is not recommended except in patients with adverse effects, so we adjusted the dosage to target trough levels of 2–5 μg/ml [3]. The patient exhibited signs of cardiogenic shock refractory to treatment, such as elevated pulmonary artery pressure, high central venous pressure, and reduced SvO2 and pulmonary artery pulsatility index. ECMO was initiated, and the patient was intubated for mechanical ventilation based on an algorithm [4]. On the 6th day, a feeding catheter and a drainage catheter were placed in the left femoral artery and vein, respectively. ECMO was maintained for 9 days. Heparin was used as the anticoagulant, and ACT levels were managed to a target range of 180–220. On the 7th day, histological examination of the myocardial biopsy led to the diagnosis of fulminant giant cell myocarditis (Fig. 3). The blood sample test showed no findings that suggested autoimmune conditions. There were no remarkable findings in contrast-enhanced computed tomography of the whole body. Histologically, there was no evidence of eosinophilic myocarditis, sarcoidosis, or hypersensitivity reactions. On the 13th day, LVEF had recovered to 27.7%, and severe mitral regurgitation was noted with improvement in RV function and absence of arrhythmias. Immunosuppressive therapy was reduced early in the clinical course, with careful balancing of the risk of disease progression against the heightened susceptibility to infection associated with immunosuppression. The IMPELLA CP was removed on the 16th day. The patient was extubated on the 20th day. However, episodes of incessant ventricular tachycardia began to increase. The patient’s persistent arrythmias and heart failure exacerbated by severe mitral regurgitation could no longer be managed solely by medication. On the 25th day, an IMPELLA 5.5 device was inserted via the right subclavian artery. On the 27th day, a recurrence of atrioventricular block was observed, and a temporary pacemaker was again inserted into the right ventricle. Considering the possibility of recurrent myocarditis, a 3-day course of intravenous methylprednisolone (500 mg daily) and tacrolimus (1.0 mg daily) were added to the immunosuppressive regimen. We adjusted the tacrolimus dosage to target a blood concentration of 10–15 ng/ml [3]. Tacrolimus was administered via nasogastric tube, but malabsorption of tacrolimus in the gastrointestinal tract was suspected because of significant gastric residual volume and persistent watery stools. Indeed, tacrolimus blood levels were low, so the tacrolimus dose was increased to maintain adequate blood concentrations. On the 41th day, the tacrolimus dose was increased from 2.5 mg to 3.0 mg, and the blood concentration rose from 3.97 ng/ml to 8.7 ng/ml. The clinical scenario was mostly consistent to one of the giant cell myocarditis in the aspects of persistent inflammation, resistance to treatment, and cardiogenic shock. Therefore, we did not repeat biopsy because the potential benefit of follow-up biopsy did not outweigh the procedural risks. Moreover, we could not administer cardiac magnetic resonance imaging because of the unstable situation including ECPELLA, for which CMR is unsuitable.

On the 34th day, the patient developed acute kidney injury associated with cardiogenic shock, and continuous renal replacement therapy (CRRT) was initiated. Concurrently, elevated inflammatory markers including C-reactive protein, white blood cells, ferritin persisted. Cytomegalovirus antigenemia was positive, and we initiated ganciclovir between day 39 and 81.

On the 48th day, heart failure was compensated for, and the IMPELLA 5.5 device was removed. On the 64th day, the patient was transferred out of the intensive care unit, and switched to intermittent renal replacement therapy. On the 82nd day, echocardiography showed that LVEF had recovered to 54%. However, the patient ultimately died on the 111th day due to septic shock from catheter-related blood stream infection.

## Discussion

We treated giant cell myocarditis by multi-modal care that included IMPELLA, ECMO, continuous renal replacement therapy and triple immunosuppressive regimen. Functional recovery of the left ventricle was achieved. The optimal treatment regimen and duration for immunosuppressive therapy for giant cell myocarditis have not been conclusively established [5]. In this case, we initially employed corticosteroids combined with mycophenolate mofetil in an effort to avoid cyclosporine-induced nephrotoxicity. Tacrolimus and mycophenolate mofetil, in addition to prednisone, might be useful for the treatment of giant cell myocarditis [3]. These data are based on cases of post-heart transplant patients. The latest Japanese guidelines for myocarditis recommend corticosteroids and cyclosporine as first line [6]. However, because our patient had low renal function, we planned to continue MMF regarding to improvements in EF, NTproBNP, and troponin T. In addition, we managed the dose of MMF targeting a serum concentration of 2–5 μg/ml due to gastrointestinal symptoms [7].

Acute myocarditis, including giant cell myocarditis, is often known to be accompanied by right heart failure treated by ECMO [8]. IMPELLA can increase cardiac output and myocardial oxygen supply while reducing myocardial oxygen demand. Additionally, with the widespread adoption of IMPELLA, some cases of survival in fulminant myocarditis have been reported with improvement of cardiac function [9, 10]. Moreover, it suggests that early initiation of IMPELLA and combination therapy with two or three immunosuppressive agents may be effective in improving survival outcomes. This patient finally died due to the infection, but the heart failure had been under control.

In conclusion, our case underscores the potential efficacy of IMPELLA for keeping the condition stable until the triple immunosuppressive regimen starts to work in managing giant cell myocarditis. This multi-modal treatment not only controlled myocardial inflammation, it also facilitated significant recovery of cardiac function.
